# The Effectiveness of Acceptance and Commitment Therapy Group Intervention (Navigator ACT) for Parents of Children With Neurodevelopmental Disabilities: A Randomized Controlled Trial

**DOI:** 10.1002/aur.70282

**Published:** 2026-05-26

**Authors:** T. Holmberg Bergman, P. Lappalainen, A. Ghaderi, T. Hirvikoski

**Affiliations:** ^1^ Department of Women's and Children's Health Pediatric Neuropsychiatry Unit, Center for Neurodevelopmental Disorders at Karolinska Institutet (KIND) Stockholm Sweden; ^2^ Habilitation & Health, Region Stockholm Stockholm Sweden; ^3^ Center for Psychiatry Research Stockholm Sweden; ^4^ Division of Clinical Psychology University of Jyväskylä Jyväskylä Finland; ^5^ Department of Clinical Neuroscience Karolinska Institutet Stockholm Sweden

**Keywords:** acceptance and commitment therapy, ADHD, autism, intellectual disability, neurodevelopmental disabilities, parenting, parenting stress, psychological flexibility

## Abstract

High levels of parental stress and psychological inflexibility are common among caregivers raising children with neurodevelopmental disabilities. Navigator ACT is a group‐based treatment developed to increase psychological flexibility and reduce the impact of stress among parents of children with various disabilities (e.g., autism spectrum disorder, ADHD, intellectual disability, acquired brain injury). This two‐arm randomized controlled trial (*n* = 137) was conducted in Sweden within outpatient disability services setting, and pre‐registered in the clinical trials register. To compare conditions, stressed and distressed parents were randomly assigned to either the ACT group (*n* = 70) or treatment‐as‐usual (TAU, *n* = 67). In the ACT group, 83.3% completed the full course of treatment. Mixed‐model linear regression analyses indicated that ACT was significantly more effective than TAU in reducing self‐reported psychological inflexibility (*p* < 0.002, *d* = 0.84) and parenting stress *(p* < 0.001, *d* = 0.38). These improvements were maintained at four‐month follow‐up. Parents in the ACT group also reported significantly greater improvements in their children's prosocial behaviors (*p* < 0.05, *d* = 0.46). However, no significant group differences were observed in levels of parental depression, anxiety, mindfulness, or child's difficulties. ACT group treatment demonstrated promising outcomes in reducing psychological inflexibility and parenting stress in a mixed group of parents of children with different neurodevelopmental disabilities, suggesting that parent support interventions do not need to be specific to the child's diagnosis.

## Summary

This study looked at whether acceptance and commitment therapy (ACT) could help stressed parents of autistic children or other disabilities increase psychological wellbeing and reduce stress. The results showed that this 5‐session group helped parents to be more aware and accepting toward thoughts and feelings related to parenting, or their child, and act flexibly in parenting situations. They also felt less stressed and more satisfied with their role as a parent. More research is needed to see how well the benefits last overtime.

## 1 Introduction

### 1.1 Parenting Stress and Psychological Inflexibility

Parenting a child with a disability is a journey through an ever‐changing landscape, both rewarding and challenging. Parenting a child with a disability impacts parents socially, psychologically, and physically, often requiring significant adjustments (Shahali et al. 2024). Parenting stress, anxiety and depression are common in families with autistic children and children with other neurodevelopmental disabilities (NDDs), for example, attention deficit hyperactive disorder (ADHD), intellectual disability (ID), acquired brain injury (ABI) (Neece and Chan 2017; Scherer et al. 2019; Shea and Coyne 2011; Theule et al. 2013). Parental stress has been defined to arise when strains and demands in parenting exceed personal and social resources (Deater‐Deckard 1998) while vulnerability for anxiety and depression increase with chronic stress (Findling et al. 2024; Scherer et al. 2019). Parent–child interactions are transactional and bidirectional; thus, parenting stress not only affects the child but is also influenced by the child's behavior (Taraban and Shaw 2018). Comorbid psychopathology and behavioral problems, common in children with NDDs, significantly increase chronic stress risk for both parents and children (Barroso et al. 2018; Emerson and Hatton 2007; Neece et al. 2012). A recent cross‐sectional study identified challenging child behaviors as the primary risk factor for parenting stress in parents of children with disabilities (Scheibner et al. 2024). Other sources of stress are the child's functional limitations, care needs and difficulties with social adjustment. Additionally, constant worry, self‐doubt, and self‐blame contribute to parental stress as do contextual factors like societal stigma and ableism (Neely‐Barnes et al. 2010).

Psychological inflexibility refers to a rigid pattern of responding to unwanted or painful internal experiences, such as thoughts and emotions, which hinder the engagement in value‐based actions (Hayes et al. 2011). Psychological inflexibility is linked to less effective parenting, poorer family relationships (Fonseca et al. 2020; Gur and Reich 2023; Moyer and Sandoz 2015), and reactive parenting marked by guilt, self‐doubt, and dissatisfaction in the parenting role (Potharst et al. 2021). High levels of psychological inflexibility have been reported among parents of children with NDDs (Lobato et al. 2022; Sairanen et al. 2018).

*Psychological flexibility (PF)*, on the other hand, has been associated with reduced stress, psychological wellbeing and adjustment in both parents and children (Leeming and Hayes 2016; Ong et al. 2024). In parenting, PF refers to the ability to be mindfully present, practice experiential‐acceptance and acceptance of the child, and persist in values‐driven actions, even when facing uncomfortable thoughts and emotions (Cheron et al. 2009; Leeming and Hayes 2016; Whittingham and Coyne 2019). Parental PF is fundamental in maintaining psychological well‐being, good parenting practices, and resilience (i.e., the ability to adapt to adversity) in challenging situations (Beeckman et al. 2019; Gur and Reich 2023; Prevedini et al. 2020). Due to developmental, emotional, and behavioral challenges, children with NDD's are highly dependent on their parents' support and resilience (Peer and Hillman 2014). This underscores the critical need to address psychological inflexibility and parental stress (Halstead et al. 2018; Hsiao 2024). However, there remains a lack of evidence‐based interventions designed to treat the psychological needs of these parents (Hirvikoski et al. 2015). Typically, parents are offered cognitive behavior therapy based short‐term counseling (Lindo et al. 2016), or parent training to manage the child's behavior (Gould et al. 2018). However, promoting parental PF more specifically may lead to strengthening of skills that support more resilient stress appraisals, such as acceptance and flexible responses to parenting‐related thoughts and emotions (Byrne et al. 2021; Hayes et al. 2011).

### 1.2 Acceptance and Commitment Therapy for Increased Psychological Wellbeing

The primary aim of acceptance and commitment therapy (ACT) is to enhance PF by cultivating skills across six interrelated and overlapping psychological processes: mindfulness, acceptance, an open stance toward internal experiences, values clarification, observing self, and commitment to flexible, values‐based actions (Hayes et al. 2009). The ACT model for parents include a notion of the developing child and the parent–child relationship (Prevedini et al. 2020). Furthermore, promoting meaningful personal activities, rest and self‐care are an important addition to the parent‐focused ACT (Ni et al. 2025).

While meta‐analyses and RCTs have established ACT as effective for treating stress and distress (A‐tjak et al. 2015; Bai et al. 2020; Konstantinou et al. 2023), research on its use to enhance parental PF is still emerging. A few existing systematic reviews and meta‐analyses have shown promising results, but they also highlight ACT for parents as a developing field (Byrne et al. 2021; Han et al. 2021; Juvin et al. 2021; Parmar et al. 2021). Han et al. reported small to moderate effects of ACT interventions on stress and depression in family caregivers, whereas Parmar et al. found no significant reductions in stress, despite improvements in parental psychological inflexibility (Han et al. 2021; Parmar et al. 2021). A systematic review by Byrne et al. evaluated 17 high/good‐quality studies involving parents of children with disabilities or chronic conditions. Of these, 16 reported significant improvements in PF, stress, and distress (Byrne et al. 2021). Another review targeted parents of autistic children and reported positive outcomes for *group‐based* ACT interventions (Juvin et al. 2021), and a meta‐analysis by Li et al. found ACT effective for parents of children with special health care needs, improving parental flexibility, depression, and anxiety, with preliminary evidence of indirect child benefits. Group formats outperformed online or individual delivery (Li et al. 2023). Despite encouraging findings, these reviews highlighted considerable heterogeneity in intervention protocols and methodological limitations, including small sample sizes. Research concerning ACT in parenting populations is still at an early stage (Juvin et al. 2021). Larger, well‐designed RCTs are needed to establish the effectiveness of ACT in this context. It is also of importance to examine different treatment formats. As to date, there is also only one meta‐analysis concerning ACT‐based group interventions (Li et al. 2023). Furthermore, we lack research on transdiagnostic ACT protocols for parents despite the apparent benefits of such an approach. Given that these parents often face common challenges and similar causes of stress, mixed groups may offer a viable alternative to diagnosis‐specific interventions, with the added benefit of improved accessibility (Byrne et al. 2021; Holmberg Bergman et al. 2023).

Navigator ACT is a newly developed *group‐based* treatment for stressed parents of children with NDDs. According to the feasibility study, the Navigator ACT was proven feasible, credible, and well‐received in the outpatient habilitation (disabilities) services context with promising preliminary results on stress and distress (Holmberg Bergman et al. 2023). Therefore, it is now of interest to assess its effectivity in a randomized controlled trial. The primary objective of the intervention is to increase parental PF and reduce the impact of stress by fostering adaptive strategies to address both internal and external stressors.

### 1.3 Aims of the Study

The aim of this study was to evaluate the effectiveness of a five‐session group‐based ACT intervention (*Navigator ACT*), delivered to stressed parents of autistic children and children with other NDDs. Specifically, we examined whether ACT, implemented within an outpatient habilitation (disability) services setting, is an effective intervention and superior to TAU in enhancing parental PF and mindfulness, and in reducing stress, depression, and anxiety. Additionally, we compared the Navigator ACT to TAU with respect to changes in children's difficulties, and strengths.

## 2 Methods

### 2.1 Trial Design and Setting

The two‐arm parallel group RCT was approved by the Regional Ethics Committee of Stockholm, Sweden (drn 2016/526‐21‐1, 2016/526‐31/1), and carried out according to the code of ethics in the Declaration of Rickham (1964), research involving human subjects (World Medical Association 2025). It was pre‐registered at the clinical trials register (clinicaltrials.gov, ID NCTO3830476), and reported according to the CONSORT guidelines (Schultz 2010). All parents signed the informed consent. The data was stored in individual Case Report Forms (CRF) separate from medical records and managed in accordance with the General Data Protection Regulation, and the Swedish Health and Medical Services Act. The collection of data and the group treatment sessions took place during 2018–2019 in seven outpatient habilitation centers (disability clinics) in two Swedish regions. The participants were blindly randomized into the ACT group, or the TAU group.

### 2.2 Participants

Participants (*n* = 137) were parents to a child(ren) 0–18 years of age with a diagnosed disability. A priori power analysis indicated that, assuming a medium between‐group effect size (Cohen's *d* = 0.50) and an alpha level of 0.05, a minimum of 42 participants per group was required to achieve a power of at least 0.90. Participants were recruited through habilitation center websites. Information about treatment and study was provided on a dedicated webpage and at an in‐person information meeting. Parents seeking treatment were screened using a structured needs and motivation interview. The inclusion criteria were: (1) a minimum age of 18 years; (2) being a parent to a 0–18‐year‐old child; (3) with a diagnosed disability or (preschool children) unspecified NDD with severe developmental delay (315.9/F89); (4) mild to severe symptoms of parental stress, depression, or anxiety, including a self‐report of minimum 4 on a visual analogue scale from 1 (no stress, depression, or anxiety) to 10 (maximum level of stress, depression, or anxiety); (5) fluency in Swedish language; (6) possibility to participate in all the treatment sessions; and (7) willingness and ability to participate in a group. When needed, a more thorough clinical assessment evaluated parents' suitability for participation. Those with severe psychiatric issues or extreme life situations were excluded and referred elsewhere. The treatment was delivered as part of ordinary services. Group leaders conducted a structured telephone interview, after which eligible parents were invited to an information meeting where they completed self‐report measures in person. No grouping was conducted based on the child's age or diagnosis; participants were invited to intake interviews in the order in which they expressed interest in participating. Only after filling out the forms did the participants receive information regarding whether they would attend the intervention in the current or next term, depending on randomization results. TAU participants continued usual services pending ACT treatment.

### 2.3 The Study Procedure and Randomization

Participants were assigned CRF codes and *blindly* block‐randomized using an online tool (https://www.random.org/lists). The randomization was done by a research coordinator who was not involved in eligibility interviews or baseline assessments and did not have the names of the patients (only codes for the eligible participants). Randomization results were sent to clinics in sealed envelopes. Clinicians and participants were blinded to group allocation (experiment or control group) during baseline assessments, but not during post‐ or follow‐up assessments. All the participants filled in the self‐rate scales in‐person and a paper form at the clinic, and the parents could ask for clarifications from experienced therapists if needed. The baseline assessment was done 0–2 weeks before the start of treatment (time‐point one, T1), 0–2 weeks after the end of the intervention (time‐point two, T2) and at follow‐up, approximately 4 months after the end of the intervention (time‐point three, T3). ACT group participants received treatment during the same term as randomization and T1 assessment. In the control condition, parents continued to receive standard care within the disability services and were offered Navigator ACT approximately 6 months later. The control group received treatments such as medication, supportive or crisis counseling, or support targeting the child's functional difficulties (e.g., challenging behaviors).

### 2.4 Navigator ACT Treatment

**FIGURE 1:**
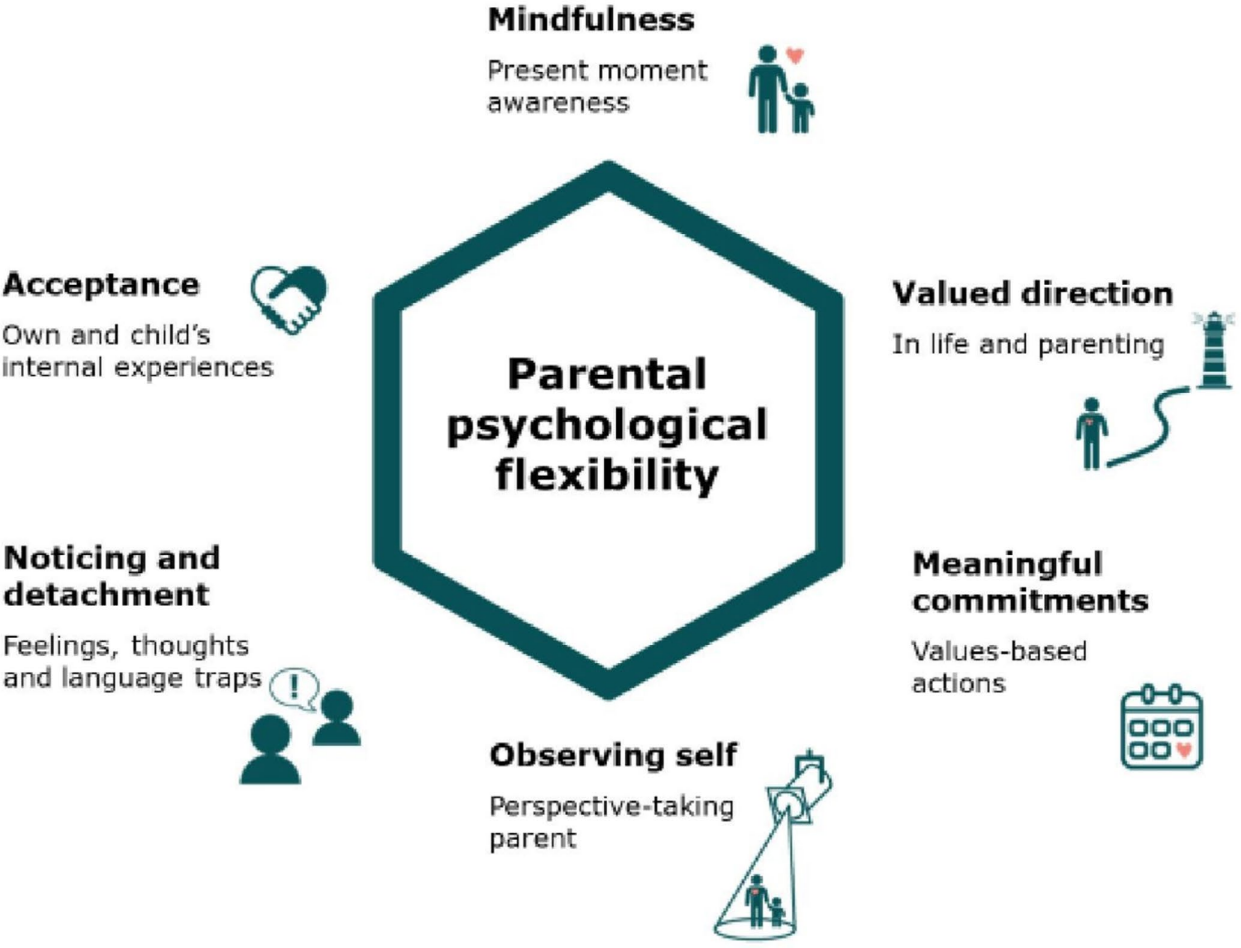
Navigator ACT treatment: parenting‐focused psychological flexibility processes. *Note:* Modified from ACT hexaflex and parent–child hexaflex (Hayes et al. 2009; Whittingham and Coyne 2019).

Navigator ACT is a context‐specific intervention developed for stressed parents of children with neurodevelopmental disorders (NDDs). It is a standardized, manualized group intervention with a structured protocol specifying the number and duration of sessions and providing example dialogues to support treatment fidelity. The program consists of five 3.5‐h sessions targeting ACT processes including acceptance, cognitive defusion, values clarification, self‐as‐context and perspective taking, and committed action in both personal and parenting domains. The aim is to increase parental PF and reduce the impact of stress through the six overlapping processes of the parent–child hexaflex model (Figure 1). Parents are also encouraged to commit to self‐care, self‐compassion, exercise, rest, and engagement in meaningful activities. A more detailed description of the sessions can be found in the previous feasibility study (Holmberg Bergman et al. 2023). The intervention was delivered in person as a closed‐group program with five 3.5‐h sessions and a 2.5‐h booster session 4 months later (total 20 h). Groups averaged 10 parents (range 6–16; recommended size 8–16). Parents received a workbook covering all exercises, and the program included experiential exercises, metaphors, role‐plays, imagined exposure, psychoeducation, and homework.

Groups were facilitated by two trained healthcare professionals, typically a licensed psychologist (*n* = 6) and a social worker (*n* = 7). One facilitator was a speech and language therapist with extensive CBT/ACT experience. All group leaders had completed at least two prior ACT groups and a six‐month ACT group leader training program including ACT theory, manual review, group facilitation skills, supervision, and clinical practice. Group leaders followed the treatment manual, and adherence was supported and monitored through supervision conducted before and after each session.

The Navigator ACT protocol was developed by a team of experienced ACT therapists, specialist psychologists, and researchers. It was informed by previous ACT protocols (Livheim 2008), an ACT group outline for parents of autistic children (Lisa Coyne, Harvard Medical School), and clinical experience and participant feedback from pilot ACT groups within disability services in Stockholm. The intervention and its feasibility are described in detail in a previous article (Holmberg Bergman et al. 2023).

## 3 Measures

### 3.1 Background, Demographic Variables and Main Outcome Measure (PAAQ)

A modified version of the *Current Life Situation Questionnaire* (Hirvikoski et al. 2009) was used to collect data on the background and clinical information of the participants, including child's diagnosis. The main outcome was measured by the *Parental Acceptance and Action Questionnaire* (PAAQ) (Cheron et al. 2009). The Swedish version of the PAAQ consists of 16‐items, and measures three dimensions of parental PF; namely, flexible action‐taking in the context of parenting, experiential acceptance of internal experiences related to parenting, and experiential acceptance of the child's internal experiences. The psychometric evaluation showed that the Swedish PAAQ exhibits good construct validity, satisfactory internal consistency, and good stability over time. The items in the PAAQ are scored from 1 = *never true* to 7 = *always true*, with a total score ranging from 16 to 112. Higher scores indicate greater inflexibility, and scores higher than 58.5 can be considered as high psychological inflexibility (Holmberg Bergman et al. 2024). In this study, Cronbach's alpha was *α* = 0.65.

### 3.2 Secondary Outcome Measures

Stress was measured by the Swedish version of the *Parental Stress Scale* (PSS), an 18‐item measure of parental stress scored from one to five (Berry and Jones 1995). The total scores range from 18 to 90. Higher scores imply greater parental stress. This version of the PSS has two subscales: lack of parental rewards and role satisfaction and demands and stressors. The psychometric evaluation of the Swedish PSS showed good validity and reliability (Lindström et al. 2024). The internal consistency for the PSS in this study was *α* = 0.81.

Symptoms of depression and anxiety were measured with the *Hospital Anxiety and Depression Scale* (HADS: Zigmond and Snaith 1983). HADS is a 14‐item rating scale (scored from 0 to 3) with two subscales measuring anxiety (HADS‐A) and depression (HADS‐D), with a total score ranging from 0 to 42. The scale is usually interpreted as separate subscales of anxiety and depression, in which case the scores of ≥ 8 out of 21 indicate clinical anxiety or depression symptomatology (Bjelland et al. 2002). HADS has good psychometric properties. The internal consistency for the total scale in this study was *α* = 0.85 (anxiety subscale *α* = 0.80, depression *α* = 0.77).

*Mindful Attention and Awareness Scale* (MAAS) was used to assess dispositional mindfulness. It is a 15‐item scale, wherein higher scores reflect greater mindful awareness. Items are scored from 1 (“almost always”) to 6 (“almost never”). MAAS has good construct validity and internal consistency over time (Brown and Ryan 2003). In the current study, MAAS showed an internal consistency of *α* = 0.87. *The Strengths and Difficulties Questionnaire* (SDQ P4‐17) was used to measure the child's difficulties (emotional symptoms, conduct problems, peer relationship problems, and hyperactivity/inattention) and strengths (prosocial behaviors) (Goodman 2001). The scores for the total score range from 0 to 40, and for the subscales from 0 to 10. Higher scores indicate a higher likelihood of difficulties or prosocial behaviors. In this study, the total score of the SDQ had an internal consistency of *α* = 0.70. For the subscales regarding difficulties, the alpha was between *α* = 0.65–67 and *α* = 0.80 (prosocial behaviors).

### 3.3 Adverse Events, Serious Adverse Events, and Harms

The group leaders were advised to register adverse events and serious adverse events during the treatment (both ACT and TAU), and during the period from T2 to follow‐up session, by following the structured instructions provided in the CRF. An adverse event was defined as any harm or negative event spontaneously reported by the patient. A serious adverse event was defined as a major difficulty that required, for example, a professional healthcare visit or hospitalization. The group leaders were also instructed to actively encourage patients during each session to report any worsening of clinical symptoms, such as depression or anxiety, throughout the study period. Both spontaneously and actively reported events and harms were registered as adverse events by the group leaders.

### 3.4 Statistical Analysis

Statistical analyses were performed using IBM SPSS Statistics version 28, alpha was set at *p* ≤ 0.05. The amount of missing data in the background and demographic data was low, ranging from 0.7% to 2.9%. If there was only one item missing in any of the self‐rating scales, the missing value was substituted with the average of the respondent's observed items.

The background and sociodemographic variables of the ACT and TAU groups were compared at baseline using independent samples *t*‐test or chi‐square test. If the difference was significant at *p* < 0.05 level, an effect size was calculated and expressed as Cohen's *d* for *t*‐test (*d* = 0.20 to 0.49 small; *d* = 0.50–0.79 moderate, and *d* > 0.80 large effect size), or Cramer's *V* for Chi Square‐test (*V* = 0.10–0.20 weak, 0.20–0.40 moderate, 0.40–0.60 relatively strong, 0.60–0.80 strong and > 0.80 a very strong association) (Cohen 1988). In addition, a post hoc ancillary analysis was conducted to examine potential differences in parental outcome measures at baseline between parents of autistic children and parents of children with other NDDs.

All participants were included in the analyses according to the intention‐to‐treat principle, using mixed‐effects linear regression models. The assumptions for Mixed Methods Linear Regression (MMLR) were met. There were few outliers, not extreme ones, which were therefore included in the analysis. The MMLR was used to examine the group differences between the two conditions, ACT intervention, and TAU, from Timepoint 1 (T1) to Timepoint 2 (T2) and T1 to Timepoint 3 (T3). We ran the model by adding *time*, *group*, and *group by time* interaction as fixed effects, and a random intercept. The unstructured covariance structure was chosen based on the lowest Akaike's Information Criterion (AIC) score. The between‐condition effect sizes and 95% confidence intervals (CIs) from the T1 to T2, and the T1 to T3 were calculated following the growth model analysis (Feingold 2015) by dividing the model‐based estimates of the mean change by the pooled (raw) standard deviation at baseline. Furthermore, the within group effect sizes for each outcome were calculated using an online template (cam.ac.uk). All effect sizes were interpreted as the Cohen's *d* as specified above (Cohen 1988).

## 4 Results

### 4.1 Background and Clinical Characteristics of the Participants

**FIGURE 2:**
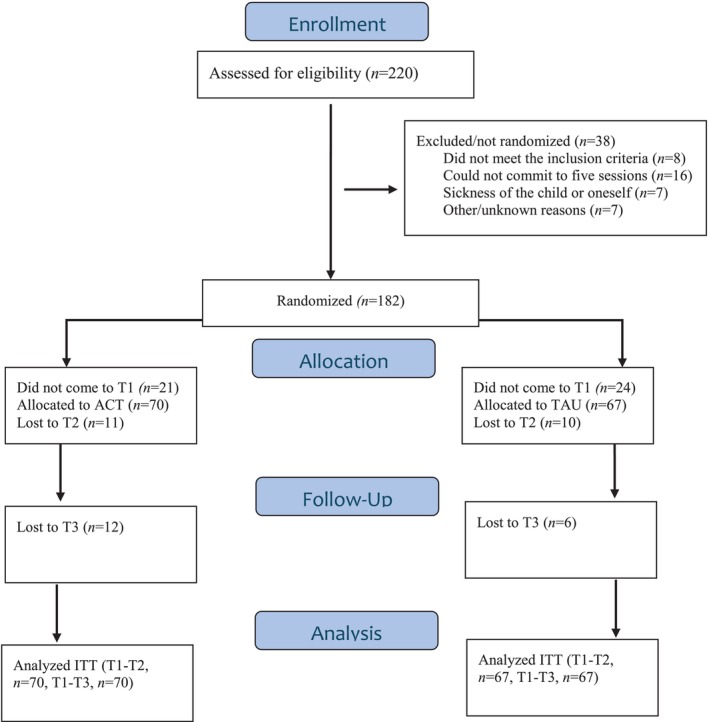
Flow‐chart of the participants. ACT = acceptance and commitment therapy, ITT = intension to treatment.

Eligible participants (*n* = 182) were randomized to ACT or TAU groups of which *n* = 137 decided to participate, ACT *n* = 70, or TAU *n* = 67 (Figure 2; Flowchart).

In the experiment group, the completion rate was 80%, that is, 56 parents out of 70 attended at least four out of five sessions (Table 1).

**TABLE 1:** Background and clinical characteristics of the parents in ACT and TAU samples.

Parent	Total *M* (SD) min–max	ACT *M* (SD) min–max	TAU *M* (SD) min–max	Test statistics *t*‐test
Age	44.5 (6.83) 26–68	43.7 (6.96) 26–59	45.3 (6.65) 30–68	n.s.
Background	*n* = 137 (100%)	*n* = 70 (51.1%)	*n* = 67 (48.9%)	Chi square *χ* ^2^
Sex/gender	116 (84.7) women	65 (85.7) women	56 (83.6) women	n.s.
Own disability	122 (91.7) none	65 (94.2) none	57 (89.1) none	n.s.
Education	81 (59.6) higher education	40 (57.1) higher education	41 (62.1) higher education	n.s.
Employment	114 (83.2) employed^a^	62 (88.6) employed^a^	52 (77.6) employed^a^	n.s.
Parenting	113 (84.3) fulltime	61 (88.4) fulltime	52 (80.0) full‐time	n.s.
Number of children	91 (66.9) 1–2	44 (63.7) 1–2	47 (70.2) 1–2 child	n.s.
Physical illness	66 (48.2) yes	39 (55.7) yes	27 (40.3) yes	n.s.
On‐going support at baseline				
Supportive counseling	25 (18.2) yes	9 (12.9) yes	16 (23.9) yes	n.s.
Duration counseling	21 (17.4) > month	8 (88.9) > month	13 (81.2) > month	n.s.
Parent training/advice	98 (77.5) yes	49 (72.1) yes	49 (84.5) yes	n.s.
Medication (Psycho pharmaceutics)	69 (37.4) yes	23 (34.3) yes	46 (40.6) yes	n.s.

*Note:* There were no significant differences between groups regarding the variables in this table (*t*‐test or chi square‐test).

Abbreviations: Child = the child with disability; TAU = treatment as usual group.

^a^ Includes employment, self‐employment, and studying.

Four parents dropped out before the first sessions, four attended only 1–2 sessions, and six attended three sessions. The participants (*n* = 137) in both groups were mainly mothers (84.7%). They parented at least one child with a diagnosed disability and were living in two parent households (73.5%) (Table 2). The majority of children were diagnosed with ASD (44.7%), ADHD (33.7%), the rest with other disabilities such as ID, CP or ABI (21.6%). Of these children, 44.5% had more than one NDD diagnoses (Table 3). Parents in both conditions reported prominent levels of psychological inflexibility (PAAQ, *M* = 67.2 ACT, *M* = 64.9 TAU) and parenting stress at baseline; 48.2% had even physical health concerns, for example, back and neck pain, migraines, and high blood pressure. Use of psychoactive drugs was reported by 37.4% of the parents. The majority (83.3%) had not changed their medication regimen in the last 3 months. No significant differences were observed between the groups at T1 with respect to sociodemographic or clinical variables.

**TABLE 2:** Background and clinical characteristics of children with disabilities.

Child	Total *M* (SD) min–max	ACT *M* (SD) min–max	TAU *M* (SD) min–max	Test statistics *t*‐test
	10.4 (4.33) 1–19	10.7 (4.45) 1–19	10.1 (4.23) 1–17	n.s.
	*n* = 137 (100%)	*n* = 70 (51.1%)	*n* = 67 (48.9%)	Chi Square *χ* ^2^
Sex/gender	88 (64.2) boys	48 (68.6) boys	34 (57.6) boys	n.s.
Disability	85 (44.7) ASD	44 (45.4) ASD	41 (44.1) ASD	—
	64 (33.7) ADHD	31 (32.0) ADHD	33 (35.5) ADHD	
	12 (6.3) ID	7 (7.2) ID	54 (5.4) ID	
	29 (15.1) Other^a^	15 (15.5) Other^a^	14 (15.1) Other^a^	
ASD vs. other disability	105 (44.7) Other^b^	53 (54.6) Other^b^	52 (55.9) Other^b^	n.s
	85 (55.3) ASD	44 (45.4) ASD	41 (44.1) ASD	
Number of diagnoses	76 (55.5) One	40 (57.1) One	36 (53.7) One	n.s.
	61 (44.5) Several	30 (42.9) Several	31 (46.3) Several	
Living	100 (73.5) two parent household	51 (72.9) two parent household	49 (74.2) two parent household	*n.s*.

Abbreviations: ADHD = attention deficit hyperactive disorder; ASD = autism spectrum disorder; Child = child with disability; ID = intellectual disability; TAU = treatment‐as‐usual.

^a^ Motor disabilities (cerebral palsy), acquired brain injury.

^b^ ADHD, intellectual disability, motor disabilities, acquired brain injury. All diagnoses are reported. As participants may have multiple diagnoses, the total exceeds the number of cases.

**TABLE 3:** The results of the MMLR for the changes in psychological inflexibility regarding ACT and TAU samples.

Primary measure	Mean (CI)			Effect size Cohen's *d* (95% CI) and results of the MMLR		
	T1 *M* (CI)	T2 M (CI)	T3 *M* (CI)	Between groups T1–T2	Between groups T1–T3	Within group T1‐T2
PAAQ‐T ACT	67.2 (63.6–70.7)	55.7 (52.0–59.4)	51.0 (46.7–55.3)	*d* = 0.84 (0.31–1.37) *B* = −8.94, *t* (233.2) = −3.03, *p* < 0.01	*d* = 0.96 (0.36–1.55) *B* = −10.24, *t* (243.5) = −3.18, *p* < 0.01	*d* = 1.54 (1.16–1.91)
TAU	64.9 (61.4–68.3)	62.3 (58.7–65.9)	60.0 (56.1–63.8)			*d* = 0.45 (0.10–0.79)
PAAQ‐A ACT	16.0 (15.1–17.0)	14.7 (13.7–15.7)	14.6 (13.5–15.7)	*d* = 0.36 (0.00–0.66) *B* = −1.36, *t* (210.8) = −2.39, *p < 0.05*	n.s.	*d* = 0.37 (0.03–0.70)
TAU	15.8 (14.9–16.8)	15.8 (14.8–16.8)	15.2 (14.2–16.3)			*d* = 0.15 (−0.19–0.49)
PAAQ‐S ACT	27.4 (26.0–28.7)	23.8 (22.4–29.0)	22.3 (20.7–23.9)	*d* = 0.54 (0.21–0.87) *B* = −2.97, *t* (218.5) = −3.24, *p* < 0.001	*d* = 0.81 (0.00–1.18) *B* = −4.47, *t* (224.7) = −4.33, *p* < 0.001	*d* = 0.98 (0.62–1.32)
TAU	25.8 (24.4–27.1)	25.2 (23.8–26.6)	25.1 (23.6–26.6)			*d* = 0.12 (−0.22–0.46)
PAAQ‐C ACT	23.8 (22.5–25.0)	21.2 (19.9–22.5)	23.7 (19.7–22.4)	*d* = 0.32 (0.06–0.58) *B* = −1.68, *t* (211.0) = −2.44, *p* < 0.05	n.s.	*d* = 0.02 (−0.31–0.35)
TAU	23.3 (22.3–24.5)	22.4 (21.1–23.7)	21.8 (20.5–23.0)			*d* = 0.32 (−0.22–0.46)

Abbreviations: ACT = acceptance and commitment therapy; CI = confidence interval; PAAQ‐A = action taking in parenting contexts; PAAQ‐C = acceptance of child's internal experiences; PAAQ‐S = acceptance of parenting related internal experiences; PAAQ‐T = Parental Acceptance and Action Questionnaire, total score; TAU = treatment‐as‐usual.

#### 4.1.1 Ancillary Analysis of Parental Outcome Variables at Baseline

We compared baseline measures of stress and mental health outcomes (PAAQ, PSS, HADS, MAAS) between parents of autistic children and those of children with other NDDs to determine whether symptom profiles differed between the ACT and TAU groups. The results indicated that there were no statistically significant differences in stress or mental health profiles between the groups at baseline.

### 4.2 Primary Outcome Measure: Psychological Flexibility

**FIGURE 3:**
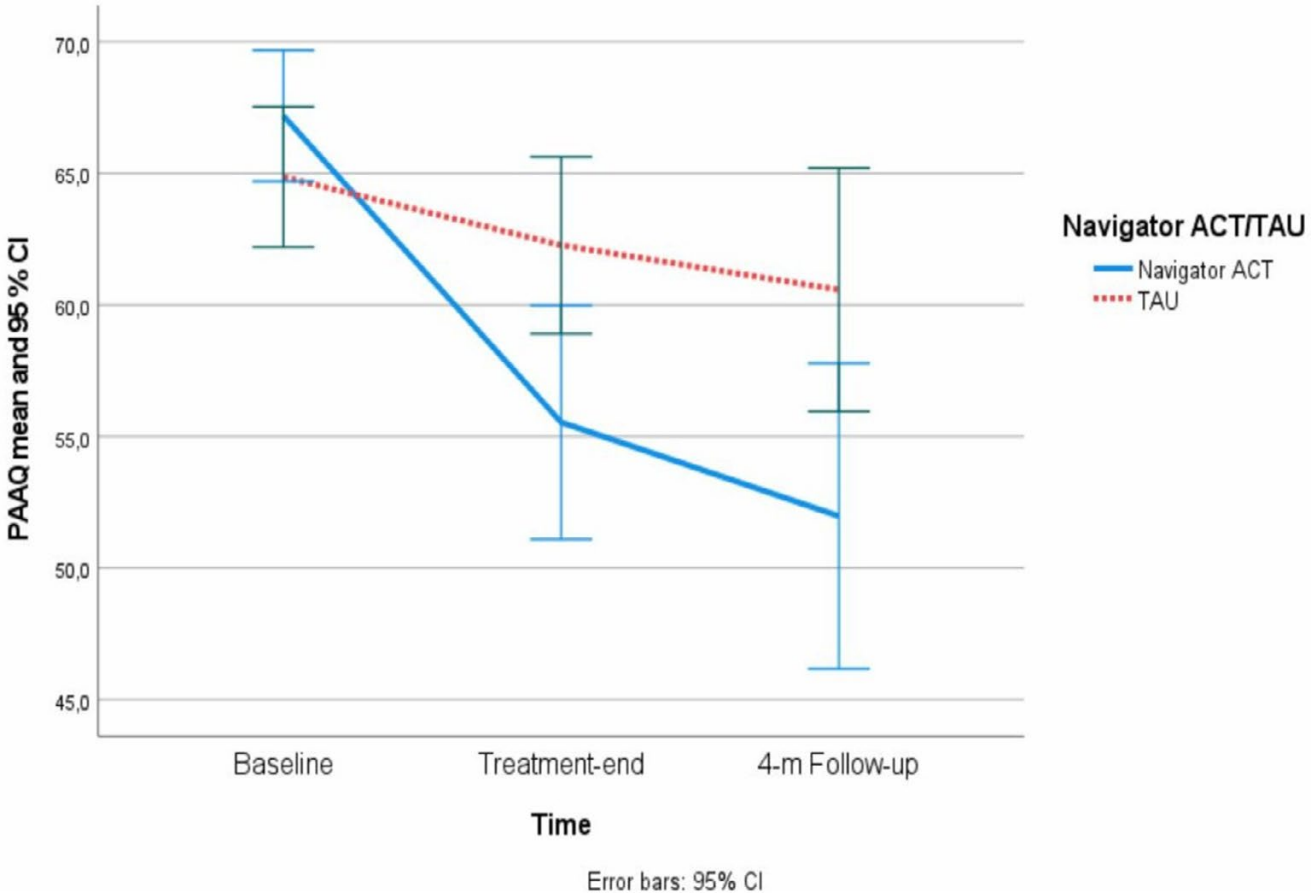
Reduction in psychological inflexibility (PAAQ scores) in ACT versus TAU groups analyzed using MMLR. *Note:* Mean scores and 95% confidence intervals (error bars) on the PAAQ at T1, T2, and T3 for the ACT group (blue solid line) and the TAU group (red dashed line).

We found a significant *group by time* interaction effect regarding the main outcome measure of parental PF (PAAQ), favoring the ACT condition, *B* = 64.9, 95% CI [61.2–68.5], *t* (285.4) =35.3, *p* < 0.001. The mean change was greater for parents in the ACT condition both from the T1 to T2 (*p* < 0.01, *d* = 0.84) and from the T1 to T3 (*p* < 0.01, *d* = 0.96) with *large* effect sizes (Figure 3). Further analysis of the PAAQ subscales revealed that the ACT treatment group demonstrated a significantly greater reduction from T1 to T2 across all three dimensions of psychological inflexibility.

The between‐group effect sizes for the three subscales were small to moderate (action taking in the parenting context, *p* < 0.05, *d* = 0.35, acceptance of own parenting related internal experiences, *p* < 0.001, *d* = 0.55, and acceptance of the child's internal experiences, *p* < 0.05, *d* = 0.32). At T3, the subscale of *acceptance of parenting related internal experiences* was significantly superior to TAU, *B* = −4.47, *t* (224.7) = −4.33, *p* < 0.001, *d* = 0.81 with a large effect size; the other two subscales were no longer significant (Table 4). The within‐group decreases in psychological *inflexibility* were as follows: from *M* = 67.2 to *M* = 51.0 in the ACT group and from *M* = 64.9 to *M* = 60.0 in the TAU group.

**TABLE 4:** The results of the MMLR for the secondary outcome measures regarding ACT and TAU samples.

Secondary measures	Mean (CI)			Effect size Cohen's *d* (95% CI) and results of the MMLR		
	T1	T2	T3	Between groups T1–T2	Between groups T1–T3	Within group T1–T2
PSS‐T ACT	46.7 (44.4–48.9)	42.8 (40.4–45.1)	43.5 (40.9–46.0)	*d* = 0.38 (0.11–0.66) *B* = −3.87, *t* (212.7) = −3.29, *p* < 0.001	*d* = 0.40 (0.16–0.40) *B* = −3.65, *t* (215.9) = −2.74, *p* < 0.01	*d* = 0.35 (0.02–0.68)
TAU	46.9 (44.6–49.2)	46.8 (44.5–49.2)	47.3 (44.9–49.0)			*d* = −0.04 (−0.37–0.30)
PSS‐S ACT	29.9 (28.3–31.5)	26.0 (24.4–27.7)	25.5 (23.6–27.4)	*d* = 0.74 (0.27–1.20) *B* = −3.60, *t* (225.2) = −3.10, *p* < 0.01	*d* = 0.75 (0.23–1.27) *B* = −3.60, *t* (225.2) = −2.84, *p* < 0.01	*d* = 0.87 (0.52–1.21)
TAU	29.8 (28.1–31.4)	29.5 (27.8–31.1)	29.1 (27.2–30.8)			*d* = 0.05 (−0.29–0.39)
PSS‐R ACT	15.4 (14.2–16.7)	14.1 (12.8–15.4)	13.5 (12.1–14.9)	*d* = 0.35 (0.16–0.81) *B* = −1.78, *t* (218.2) = −2.40, *p* = 0.017	*d* = 0.48 (0.06–0.48) *B* = −2.44, *t* (222.9) = −2.94, *p* < 0.01	*d* = 0.35 (0.02–0.69)
TAU	15.2 (14.0–16.5)	15.7 (14.4–17.0)	15.7 (14.4–17.1)			*d* = −0.11 (−0.44–0.23)
HADS‐T ACT	20.2 (14.1–17.7)	16.4 (14.7–18.1)	15.9 (14.1–17.7)	n.s.	n.s.	*d* = 0.70 (0.35–1.04)
TAU	21.9 (20.2–23.5)	18.1 (16.4–19.8)	18.4 (16.6–20.3)			*d* = 0.50 (0.15–0.84)
HADS‐A ACT	12.0 (11.0–12.9)	9.9 (8.9–10.9)	9.5 (8.5–10.7)	n.s.	n.s.	*d* = 0.66 (0.32–1.00)
TAU	12.9 (11.9–13.9)	10.6 (9.6–11.6)	11.0 (9.9–12.2)			*d* = 0.46 (0.11–0.80)
HADS‐D ACT	8.2 (7.3–9.1)	6.5 (5.6–7.3)	6.3 (5.4–7.2)	n.s.	n.s.	*d* = 0.60 (0.25–0.93)
TAU	9.0 (8.1–9.8)	7.5 (6.6–8.4)	7.5 (6.5–8.4)			*d* = 0.40 (0.06–0.74)
MAAS ACT	3.30 (3.1–3.5)	3.58 (3.4–3.8)	3.55 (3.3–3.8)	n.s.	n.s.	*d* = 0.39 (−0.73–0.06)
TAU	3.13 (2.9–3.3)	3.36 (3.1–3.6)	3.55 (3.3–3.8)			*d* = 0.56 (0.21–0.90)

Abbreviations: ACT = acceptance and commitment therapy; CI = confidence interval; HADS‐A = anxiety; HADS‐D = depression; HADS‐T = Hospital Anxiety and Depression Scale; MAAS = Mindfulness Acceptance and Awareness Scale; PSS‐R = lack of rewards and role satisfaction; PSS‐S = stressors; PSS‐T = parental stress scale total score; TAU = treatment‐as‐usual.

### 4.3 Secondary Outcome Measures

**FIGURE 4:**
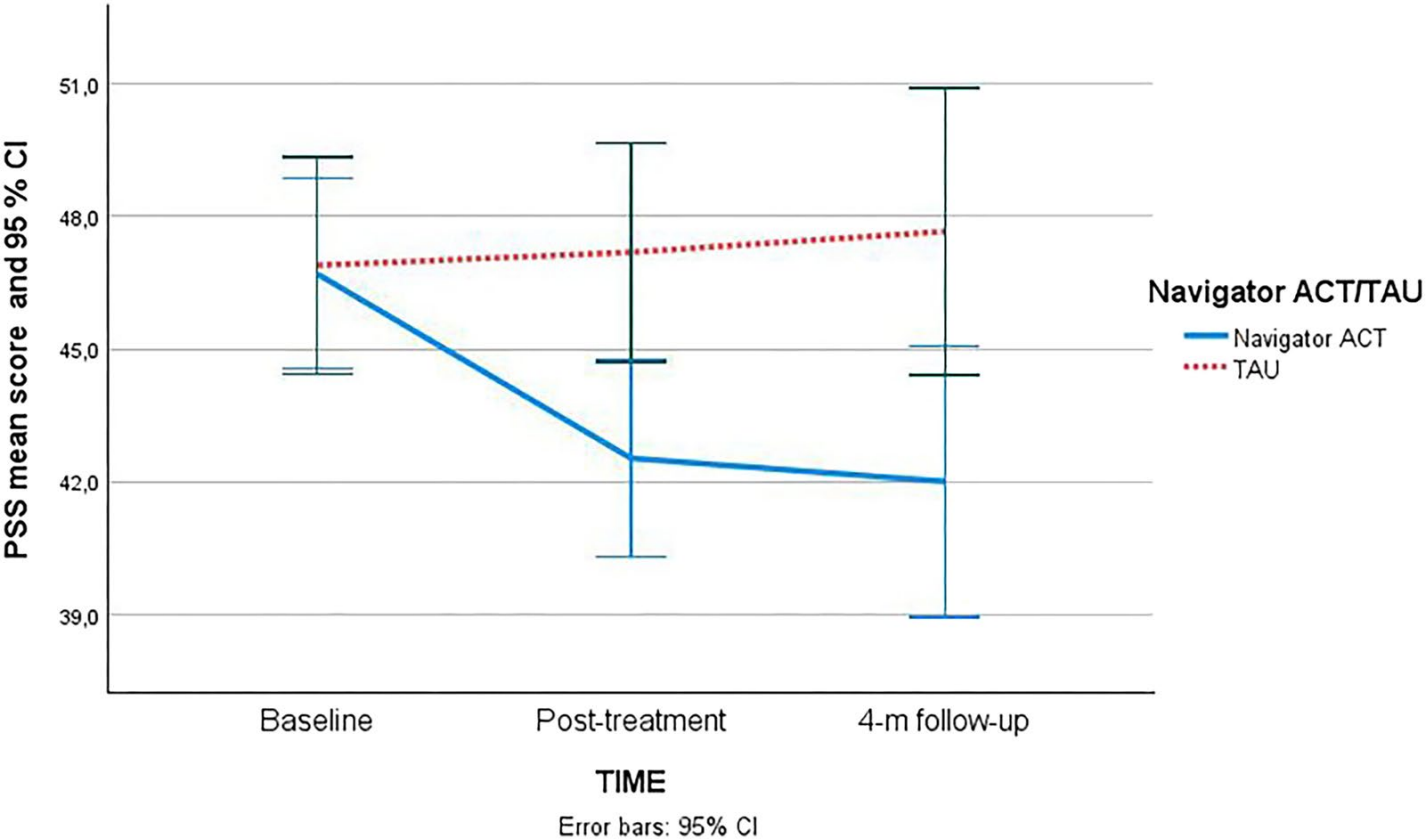
Reduction in parental stress (PSS scores) in ACT versus TAU groups analyzed using MMLR. *Note:* Mean scores and 95% confidence intervals (error bars) on the PSS at T1, T2, and T3 for the ACT group (blue solid line) and the TAU group (red dashed line).

There was a statistically significant *group by time* interaction regarding parenting stress (PSS), *B* = 46.9, 95% CI [44.6, 49.2], *t* (176.7) = 39.7, *p* < 0.01, favoring the ACT condition. The difference was significant both from the T1 to T2 (*p < 0.05*, *d* = 0.38, 95% CI 14.7–3.13), and from the T1 to T3 (*p* < 0.05, *d* = 0.35, 95% CI 16.6–3.89) with small effect sizes (Figure 4). When the subscales were analyzed separately, the decline in parenting stressors (*p* < 0.05, *d* = 0.57, 95% CI 5.90–1.31) showed medium effect size, while the effect size for the lack of rewards/role satisfaction was small (*p* < 0.01, *d* = 0.35, 95% CI 4.07–0.80). These effect sizes increased slightly by T3, *p* < 0.05, *d* = 0.58 and *p* < 0.05. *d* = 0.48, respectively (Table 4). In addition, there was an interaction effect regarding the positive change in the child's prosocial behaviors. The ACT condition was superior to the TAU, *B* = 6.1, 95% CI [5.5, 6.8], *t* (288.1) = 19.8, *p* < 0.05 (Table 5). The effect size from T1 to T2 was small (*p* < 0.05, *d* = 0.45, 95% CI [5.9,1.31]), and from the T1 to T3 medium (*p* < 0.05, *d* = 0.51, 95% CI [6.23–1.13]). We did not find significant differences regarding the child's difficulties, parental depression, anxiety, or mindfulness.

**TABLE 5:** The results of the MMLR for the child‐related secondary outcome measures.

Secondary measure	Mean (CI)			Effect size Cohen's *d* (95% CI) and results of the MMLR		
	T1	T2	T3	Between treatments T1–T2	Between treatments T1–T3	Within group T1–T2
SDQ‐T ACT	19.4 (18.0–20.7)	17.6 (16.2–19.1)	17.3 (15.6–19.0)	n.s.	n.s.	*d* = 0.38 (0.05–0.72)
TAU	18.3 (17.0–19.7)	18.1 (16.7–19.6)	17.8 (16.2–19.4)			*d* = 0.09 (−0.25–0.43)
SDQ‐E ACT	4.8 (4.2–5.5)	4.2 (3.5–4.9)	4.2 (3.5–5.1)	n.s.	n.s.	*d* = 0.24 (−0.09–0.58)
TAU	4.5 (3.9–5.2)	4.4 (3.7–5.1)	4.4 (3.7–5.1)			*d* = 0.04 (−0.20–0.48)
SDQ‐C ACT	3.6 (3.1–4.1)	3.2 (2.7–3.8)	3.0 (2.4–3.6)	n.s.	n.s.	*d* = 0.27 (−0.06–0.60)
TAU	3.3 (2.8–3.8)	2.9 (2.4–3.4)	3.0 (2.4–3.5)			*d* = 0.09 (−0.20–0.48)
SDQ‐H ACT	6.5 (5.9–7.1)	6.0 (5.4–6.6)	6.1 (5.4–6.8)	n.s.	n.s.	*d* = 0.16 (−0.17–0.49)
TAU	6.5 (5.9–7.1)	6.4 (5.8–7.0)	6.3 (5.6–6.9)			*d* = 0.09 (−0.25–0.43)
SDQ‐P ACT	4.5 (4.0–5.1)	4.2 (3.6–4.8)	4.0 (3.2–4.7)	n.s.	n.s.	*d* = 0.21 (−0.12–0.54)
TAU	4.0 (3.5–4.6)	4.4 (3.8–5.0)	4.2 (3.6–4.9)			*d* = −0.08 (−0.42–0.26)
SDQ‐PR ACT	5.3 (4.7–5.9)	5.9 (5.3–6.6)	6.1 (5.3–6.8)	*d* = 0.46 (0.04–0.88) *B* = 1.166, *t* (215.1) = 2.17, *p* < 0.05	*d* = 0.53 (0.05–0.88) *B* = 1.337, *t* (228.6) = 2.25, *p* < 0.05	*d* = 0.32 (0.02–0.65)
TAU	6.2 (5.6–6.8)	5.7 (5.1–6.3)	5.7 (5.0–6.4)			*d* = 0.15 (−0.19–0.49)
SDQ‐I ACT	5.4 (4.7–6.0)	4.9 (4.2–5.7)	4.9 (4.2–5.7)	n.s.	n.s.	*d* = 0.19 (−0.14–0.52)
TAU	5.2 (4.5–5.8)	5.1 (4.4–5.8)	5.1 (4.4–5.8)			*d* = 0.03 (−0.30–0.37)

Abbreviations: ACT = acceptance and commitment therapy; CI = confidence interval; SDQ‐C = conduct difficulties; SDQ‐E = emotional difficulties; SDQ‐H = hyperactivity difficulties; SDQ‐I = the impact score; SDQ‐P = peer difficulties; SDQ‐PR = prosocial behaviors; SDQ‐T = Strengths and Difficulties Questionnaire; TAU = treatment‐as‐usual.

### 4.4 Adverse and Serious Adverse Events or Harms

No *serious* adverse events were reported in either group. However, six adverse events were spontaneously reported by the patient, primarily related to a worsening of the child's symptoms or challenging periods, such as ongoing toilet training. Additionally, one participant verbally reported a worsening of depressive and anxiety symptoms to a group leader at follow‐up.

## 5 Discussion

### 5.1 Main Findings

This study evaluated the effectiveness of a five‐session ACT group treatment for parents raising children with NDDs. The Navigator ACT group intervention was significantly more effective than TAU in enhancing PF. Additionally, it outperformed TAU in reducing parenting stress and increasing parent‐reported prosocial behaviors in children. However, the ACT intervention showed no greater effectiveness than TAU in improving symptoms of anxiety, depression, or mindfulness. The overall completion rate of 80% was in line with our earlier feasibility study (Holmberg Bergman et al. 2023), and what has been previously reported (80%–84%) in ACT interventions (Karekla et al. 2019). However, it was lower than the 25%–60% that has been reported in connection with parenting interventions (Chacko et al. 2016).

Parental PF is a key component of psychological health (Gur and Reich 2023; Kashdan and Rottenberg 2010), and mediates reductions in stress, and improvements in parental resilience as well as parenting practices. Parental inflexibility has been associated with harsh or overly passive parenting styles, which negatively impact child development, behavior, and the parent–child relationship (Fonseca et al. 2020). This study demonstrated that the ACT group intervention enhanced parental PF, especially experiential acceptance of internal experiences, such as thoughts and feelings related to parenting. These findings align with recent systematic reviews which highlighted ACT as a promising approach for increasing parental PF and well‐being (Byrne et al. 2021; Han et al. 2021; Juvin et al. 2021; Li et al. 2023). More specifically, previous research has shown improvements in parental PF following ACT in parents of autistic children (Fung et al. 2018; Hahs et al. 2019), and in children with other chronic conditions (Çiçek Gümüş and Öncel 2023; Lappalainen et al. 2021; Sairanen et al. 2019). While earlier studies have primarily focused on parents of children with specific disabilities, this study demonstrated the positive effects of ACT in a context where parents of children with various NDDs were treated within the same group. In these groups, the focus was on the parenting experience, not diagnosis of the child. Mixed parent groups may promote greater feasibility than diagnosis‐specific groups, particularly in rural settings or when supporting families of children with rare conditions, by expanding access to intervention and support.

The improvements in parental PF observed in the ACT group remained significant at the 4‐month follow‐up, aligning with previous research demonstrating the sustained benefits of ACT interventions over time (Østergaard et al. 2020; Sairanen et al. 2019). However, a four‐month follow‐up represents a relatively short time frame. Investigating the long‐term maintenance of ACT‐related outcomes remains a critical direction for future research (Byrne et al. 2021; Lappalainen et al. 2024). Future research could also benefit from exploring long‐term effects also through qualitative feedback from the service‐users (parents) to assess the broader impact of ACT on parents' wellbeing and parenting practices.

### 5.2 Secondary Measures

In this study, ACT condition was significantly more effective than TAU in reducing parenting stress, with moderate effect sizes. As the PSS comprises two distinct dimensions, these results reflect not only reduced impact of stressors and demands but also enhanced parental well‐being through increases in positive parenting experiences. Specifically, parents reported greater perceptions of personal resources, such as rewards and role satisfaction, suggesting that changes in parenting stress encompassed both an alleviation of burden and an enhancement of positive aspects of parenthood.

Higher levels of PF have been associated with lower levels of stress (Fonseca et al. 2020; Moyer and Sandoz 2015). The findings from our study are consistent with prior research indicating the positive impact of ACT interventions on reducing stress in parents of children with NDDs (Chua and Shorey 2022; Flujas‐Contreras et al. 2024; Han et al. 2021; Kiani et al. 2025). Previous research suggests that the strength of ACT lies in its capacity to modify individuals' stress responses during and following triggering events, thereby contributing to an overall reduction in perceived stress (Frögéli et al. 2016). In line with earlier findings (Gur and Reich 2023; Barroso et al. 2018), parents in the present study reported elevated levels of psychological inflexibility and parenting stress at baseline, which both were significantly reduced following participation in the ACT intervention. Future research should further examine the relationship between parental stress and PF, as well as the potential implications for parenting practices and child developmental outcomes.

There were no significant differences between groups in the improvement of symptoms of anxiety or depression, mindfulness, or child's difficulties. These findings contrast with results from our previous *Navigator ACT* feasibility study (Holmberg Bergman et al. 2023) as well as with several other studies reporting positive effects in these domains (Byrne et al. 2021; Parmar et al. 2021).

These results regarding psychiatric symptoms may be partly explained by the fact that the trial was conducted within routine disability services. In Sweden, anxiety and depression are typically treated within psychiatric services, which meant that the primary targets of this intervention were PF and stress. Moreover, baseline severity and methodological factors could have contributed to the findings. Parents entered the study with very low depressive symptoms, leaving little room for improvement and making between‐group differences unlikely. Although anxiety symptoms were moderate at baseline and the Navigator ACT group showed a large within‐group reduction (from moderate to mild levels), TAU participants also improved, reducing the potential for between‐group contrasts. This pattern, combined with the sample size and the conservative nature of mixed‐effects modeling, limits the power to detect small incremental effects even when effect sizes favor the intervention. Consistent with previous ACT research, proximal processes such as PF show more distinct group differences, whereas distal outcomes like anxiety and depression tend to improve across both conditions unless baseline distress is elevated.

Parents in the ACT group reported significantly greater improvements in their child's *prosocial behaviors* (SDQ‐P) than those in TAU. Unfortunately, the current study did not allow for an analysis of potential mechanisms of change. Parental PF has been linked to increases in children's prosocial behavior, likely through modeling calm, sensitive, and mindful responses (Leeming and Hayes 2016; Whittingham 2014). However, *child difficulties* in SDQ‐P showed only small declines in both groups at T2 and T3 and the results did not differ significantly between groups. The Navigator ACT group demonstrated a moderate within‐group effect (*d* = 0.38), whereas TAU showed no change (*d* = −0.09), indicating some clinical improvement despite the absence of between‐group differences. Baseline conduct problems were not markedly elevated in this sample, suggesting that these children may have had fewer challenging behaviors than typically reported in NDC samples (Neece et al. 2012; Scheibner et al. 2024). Furthermore, internal consistency for the SDQ‐P was only acceptable for most subscales (*α* = 0.65–0.67), whereas the prosocial scale showed good reliability (*α* = 0.80). The relatively low internal consistency may have limited the ability to detect significant effects in some of the SDQ subscales. The use of measures with stronger psychometric properties in this population may be beneficial in future studies. The SDQ conduct subscale may also fail to capture behavior patterns common in NDCs (Emerson 2021). For example, items such as lying or stealing may be less relevant for autistic children who often show literal communication and rule‐following tendencies. Alternative measures, such as the Child Behavior Checklist, may be more appropriate for assessing behavioral difficulties in children with NDD's.

The lack of positive changes in mindfulness is puzzling, but not unusual. According to recent meta‐analysis, ACT interventions on mindfulness have yielded inconclusive results (Chua and Shorey 2022). It has been suggested that measurable improvements in mindfulness may require more prolonged and consistent practice. For example, increased awareness of mind‐wandering (an early outcome of mindfulness training) may paradoxically lead individuals to perceive a decline in their mindfulness skills (Corti et al. 2018; Singh et al. 2006).

### 5.3 Strengths and Limitations

The study's strengths include its RCT design and recruitment of participants and group leaders from publicly funded outpatient habilitation centers, enhancing the generalizability of results to similar settings. The use of robust psychometric measures (PAAQ, PSS), evaluated in large samples of parents of children with and without disabilities in Sweden, further strengthens the study (Holmberg Bergman et al. 2024). In addition, the investigation of a transdiagnostic group intervention provided new, valuable information on the possibility of offering ACT to parents of children with different types of disabilities in same groups.

This study had limitations due to the representativeness of the sample as most of the participants were highly educated (59.6% with a university degree). However, representativeness corresponds somewhat well to the population statistics of urban cities such as Stockholm where 49% of the population have completed at least 3 years of higher education (SCB, S. S 2023). Secondly, as our study was conducted within outpatient habilitation (disability) services, the patients in need of more intensive psychiatric care were referred to by other services, which affected the selection of the participants. Further investigation into the effectiveness of transdiagnostic ACT‐based treatments across diverse populations and settings is warranted, along with studies examining their impact on child well‐being. A limitation of the study was also the lack of blinding at follow‐up. Both participants and individuals involved in data collection were aware of group allocation, and outcomes relied on self‐report measures, which may have increased the risk of response biases such as social desirability or expectancy effects. This may have contributed to an overestimation of intervention effects. Furthermore, the relatively low internal consistency of some measures was a limitation in this study. The PAAQ showed an alpha below *α* = 0.70, and the SDQ consistency *α* = 0.70 (total scale). This is somewhat expected as both instruments assess broad constructs using only a few items (PAAQ; PF with six separate fast overlapping processes, and SDQ; heterogeneous domains of child functioning). Previous work of PAAQ including a psychometric evaluation in this target group with more than 2000 parents demonstrated that the PAAQ has good construct validity, adequate internal consistency, and strong test–retest reliability (Holmberg Bergman et al. 2024; Moyer and Sandoz 2015). Moreover, we retained SDQ because it is widely used, well validated, and has shown similar reliability in studies of children with NDDs, where diverse symptom profiles may affect performance (Dahlberg et al. 2020; Emerson 2021; Grasso et al. 2022; Malmberg et al. 2003). Although another measure might have captured certain behavioral changes more sensitively, the prosocial subscale (*α* = 0.80) did show improvement. In general, measures should be excluded when reliability falls below accepted thresholds (typically < 0.60) and when there is no strong conceptual or empirical rationale for retaining them. In this study, the conceptual relevance of PAAQ and SDQ, their use in earlier project phases, and their established application in related research supported their inclusion despite the limitation of lower internal consistency.

Future research would benefit from comparing Navigator ACT with an active control condition rather than TAU. Although participants had comparable access to healthcare services at baseline of the present study, patterns of service use were not monitored over the course of the trial. In addition, detailed information on the specific components of TAU—such as the type and content of counseling or child‐related support—was not collected. Nevertheless, comparison with TAU more closely reflected routine clinical practice, which is consistent with the design of effectiveness studies. Finally, the use of blinded assessors at both pre‐ and post‐intervention assessments, rather than restricting blinding to baseline only, could further reduce the risk of expectancy or placebo‐related effects.

### 5.4 Clinical Applications

The group‐based ACT interventions may benefit from closer monitoring and measurement of mindfulness skill development throughout the treatment process, with increased emphasis on mindful parenting instead of only dispositional mindfulness. Additionally, it may be advantageous to offer some parents structured parent training shortly after completing an ACT intervention. This could create opportunities to apply behavioral change strategies grounded in experiential acceptance, thereby supporting the maintenance of values‐based parenting practices, even in the face of challenging situations. In addition, to participate in parent training enhanced with ACT may represent a valuable treatment option for certain families (Gould et al. 2018; Whittingham et al. 2016). Recent studies utilizing such combined approaches have reported significant positive outcomes, not only in enhancing parental PF and self‐efficacy, but also in reducing emotional and behavioral difficulties in children (Ni et al. 2025).

In conclusion, the transdiagnostic, group‐based Navigator ACT intervention for parents of children with NDDs was effective in enhancing parental PF and reducing parenting stress. These improvements may have also positively impacted child well‐being, as indicated by parent‐reported increases in prosocial behaviors.

## Funding

This work was supported by Forskningsrådet om Hälsa, Arbetsliv och Välfärd, 2019/2020‐01665; Region Stockholm, FOUI‐951447; Stiftelsen Sunnerdahls Handikappfond; Swedish Association of Local Authorities and Regions. Stiftelsen Clas Grochinsky Minnesfond; the Swedish Order of Freemasons; Stiftelsen Promobilia; Karolinska Institutet.

## Conflicts of Interest

TatjaHirvikoski, Ata Ghaderi and Tiina Holmberg Bergman receive royaltiesfrom publisher (Hogrefe, Natur och Kultur, Studentlitteratur) for textbooks and manualsunrelated to this study. The rest of the authors had no conflicts of interest.

## Data Availability

The data that support the findings of this study are available on request from the corresponding author. The data are not publicly available due to privacy or ethical restrictions.
